# Triple negative breast cancer in a poor resource setting in North-Western Tanzania: a preliminary study of 52 patients

**DOI:** 10.1186/1756-0500-7-399

**Published:** 2014-06-26

**Authors:** Peter Rambau, Nestory Masalu, Kahima Jackson, Philipo Chalya, Patrizia Serra, Sara Bravaccini

**Affiliations:** 1 Pathology Department, Catholic University of Health and Allied Sciences-Bugando, Box 1464, Mwanza, Tanzania; 2 Department of Oncology, Bugando Medical Center, Box 1370, Mwanza, Tanzania; 3 Department of Pathology, Bugando Medical Center, Box 1370, Mwanza, Tanzania; 4 Department of Surgery, Bugando Medical Center, Box 1370, Mwanza, Tanzania; 5 IRCCS - Istituto Scientifico Romagnolo per lo Studio e la Cura dei Tumori (IRST), 47014 Meldola, (FC), Italy

**Keywords:** Brest cancer, Triple negative, North-Western Tanzania

## Abstract

**Background:**

Breast cancer is the second leading cancer worldwide. In Tanzania, though it ranks as the second leading cancer in women after cervical cancer, hormonal receptor status is not carried out routinely in patients. Adjuvant hormonal therapy is given without prior knowledge of hormonal receptors status and patients can incur unnecessary costs and side effects. This study was performed to investigate the expression of hormonal receptors, epidermal growth factor receptors (HER-2) and proliferation index of the breast cancer by Ki-67 in a few selected patients with breast cancer at referral hospital in North-Western Tanzania. The study classified breast cancer subtypes based on hormonal receptors status and the expression of epidermal growth factor receptors.

**Results:**

A total of 52 cases of breast cancer were investigated. Patients’ mean age at diagnosis was 49 years. The majority of the tumors was invasive ductal carcinoma 47 (90.4%) and 40 (76.9%) were of histological grade III. Thirty-eight (73.1%) of the patient had lymph node metastasis at the time of diagnosis and 36 (69.2%) were at clinical stage III. Only 3 (5.8%) patients were in clinical stage I. There was a tendency of a low level of expression of the receptors, whereby Estrogen Receptor (ER) positive tumors were 17 (32.7%), progesterone receptor (PR) positive tumors were 22 (42.3%), and HER-2 positive tumors were 12 (23.1%). Triple negative tumors constituted 20 (38.4%) of the patients. Most of the tumors (75%) showed high proliferation by Ki-67. Lymph node metastasis was more common in Triple Negative and HER enriched tumors.

**Conclusion:**

This study showed a tendency for a low level of expression of hormonal receptors. There was a significant proportion of Triple Negative breast cancers. Routine testing for hormonal receptors in breast cancer is recommended before the initiation of adjuvant hormonal therapy.

## Background

Testing for Estrogen (ER) and Progesterone (PR) receptors expression for breast cancer patients are routinely done in many parts of the world, as determination of estrogen and progesterone receptors expression determines the patients who will benefit from adjuvant hormonal therapy. In addition, testing of HER-2 is performed to select patients who will benefit from Trastuzumab therapy. The use of all the three markers allows the classification of patients with breast cancer in different categories with different tumor types, treatment and prognosis [1,2].

Several studies have shown that the benefit from hormonal therapy is proportional to the hormonal receptors levels [3]. HER-2 is an oncogene which belong to a family of epidermal growth factors and is amplified in 14-25% of cases of breast cancers [4-6]. Amplification for this gene leads to the expression of a trans-membrane protein which can be detected by immunohistochemistry. The current management of the breast cancer involves the use of Trastuzumab for patients’ with amplification of this gene [5]. In poor resource settings, these markers are not routinely tested and it is therefore impossible to select the patients who will benefit from adjuvant therapy. Currently there is a need to introduce routine testing of these markers in a poor resource settings as a mode of therapeutic selection in patients with breast cancer, and the status of these markers should be investigated in different regions of Africa [7,8]. In Tanzania, one study evaluated 60 cases of breast cancer for PR and ER status, where the trend was poor expression of these markers with only 26.7% the patients were expected to benefit from hormonal therapy. The patients were of young age with an advanced stage at the time of presentation [8]. The same trend was observed in Kenya where the patients had an advanced stage of the disease with a low percentage likely to be hormonal sensitive in all stages of the disease [9]. In those studies HER-2 was not tested. However, one study showed a high level of HER-2 over-expression (20.26%) and was common in Grade III invasive ductal cancers [7]. Other studies have shown a higher rate of HER-2 gene amplification in ductal cancers compared to lobular cancers; those tumors had a high histological grade with negative ER and PR status [6,10].

With regard to age, studies have shown the trend of negative receptor status at a young age. Women over 40 years are more likely to benefit from adjuvant endocrine therapy with low recurrence, whereas younger women have a high prevalence of HER-2 over expression and a low 5-years survival rate [11]. The expression of ER, PR and HER-2 is used to describe breast cancer groups which need different modalities for systemic treatment and it can predict the prognosis of the patients. Tumors which do not express PR, ER nor HER-2 are called triple-negative tumors (TN), whereby such patients do not benefit from adjuvant therapy and are left with the only option of chemotherapy and radiotherapy [5,12]. Triple negative cancers account for 10-17% of all breast cancers [5,13,14]. One study found a prevalence of 29.3% TN tumors in African American women and of 13.0% in non-African American women [15]. TN breast cancers appear common in pre-menopausal young African American women [12,15] who tend to have an aggressive form of disease presenting as a large tumor of grade III with pushing margins. The tumors tend to recur and generally they tend to have a poor outcome and a low overall survival compared to other tumors. The metastatic patterns of these tumors tend to be through blood and commonly involve lungs, brain and meninges [5,12-14]. This study presents a preliminary analysis of 52 cases of breast cancer for these markers in North-Western Tanzania. These markers have not been studied yet in this particular setting, where these tests are not routinely done.

## Methods

This was a retrospective study done at the Bugando Medical Centre (BMC). BMC is a consultant and teaching hospital serving a population of 13 million people in North-Western Tanzania, where all diagnoses of breast cancer are made. Fifty-two formalin-fixed and paraffin-embedded tissue blocks were randomly selected from archival materials at the department of pathology. Previous histology reports and patients’ files from the hospital records were used to obtain demographic data and clinical information. Histology slides were re-evaluated for histological type and the histological grade was established by the Modified Bloom-Richardson score system which scores for tubular formation, nuclear pleomorphism and mitotic rates within tumor cells. The clinical stage of the disease was determined by using TNM (AJCC cancer staging manual). This is a staging system which measures the anatomical extent of disease based on the extent of a primary tumor (T), the absence or presence of and the extent of regional lymph node metastasis (N) and the absence or presence of distant metastasis. Hormone receptors status, Estrogen Receptor, Progesterone Receptor and Ki-67 were determined by ImmunoHistoChemistry (IHC) using Dako Antibodies and Dako Autostainer (Dako, CA) at the laboratory of the Scientific Institute of Romagna for the study and treatment of Tumors (IRST) in Italy.

### Immunohistochemistry

Four-micrometre sections of the tissue samples were mounted on positive-charged slides (BioOptica, Milan, Italy). Deparaffinization was carried out by xylene and sections were rehydrated, followed by endogenous peroxidase activity block by 3% hydrogen peroxide solution. Antigens unmasking was performed by using citrate buffer pH 6 for 40 minutes at 98.5°C.

Mouse monoclonal anti-human antibodies for Estrogen receptor ER (clone-NCL-L ER-6 F11) and Progesterone receptor PR (clone NCL-L-PGR) from Novocastra, Newcastle upon Tyne, England were used at the dilution of 1:80, 1: 40 respectively. For Ki-67 and HER-2, a Mouse monoclonal anti-human antibodies (Dako, Carpinteria, California, USA clone MM1) at dilutions of 1:100 was used. Antibodies were incubated on the sections for 60 minutes at room temperature. The sections were then washed with Phosphate Buffered Saline (PBS) and incubated with a universal biotinylated secondary antibody and later on rinsed in PBS. This was followed by incubation with streptavidin-peroxidase conjugate (LSAB + Kit; DAKO Corporation, Carpinteria, CA, USA) for 15 minutes. Sections were rinsed again in PBS and antibody binding was detected by staining with diaminobenzidine/hydrogen peroxidase chromogen solution (DAB + liquid substrate–chromogen solution; DAKO Corporation). Finally, the sections were rinsed in deionized water, counterstained by Mayer’s Hematoxylin, and mounted by Eukitt (Bio-Optica).

**Figure 1:**
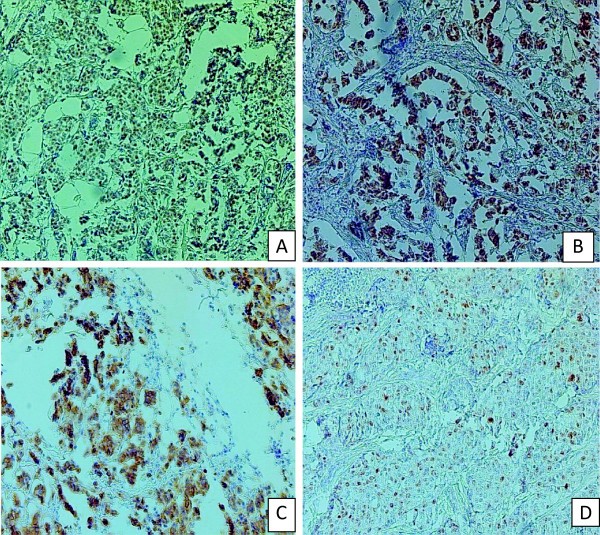
**Immunuhistochemical staining pattern. A**: Nuclear staining for ER, **B**: Nuclear stain for PR, **C**: Membrane stain for HER-2 and **D**: Nuclear staining for Ki-67.

The samples reactivity was evaluated by light microscopy (× 200) by two independent observers. The markers positivity was evaluated in a semi-quantitative way. For ER and PR, the staining was evaluated as follows: no nuclear staining was scored as 0 (Negative for receptor), nuclear staining of less than 10%, as +1(borderline), staining between 10-75% as +2 and +3 when the staining was more than 75%. In this study, the presence of nuclear staining of 10% and above +2 and +3 was sufficient to classify the cases as positive. For Ki-67, the number of stained cells against the counted tumor cells was expressed as a percentage and the staining of 10% or more of tumor cells was sufficient criteria to classify the cases as positive. Staining for HER-2 was considered as score 0 or negative when no staining was observed or when membrane staining was less than 10% of the tumour cells. A score of +1 was also considered negative, when showing a faint membrane staining in less than 10% of the tumor cells. A score +2 was considered as a weak positive when showing a weak to moderate complete membrane staining in more than 10% of the tumor cells. This was also considered as negative. A score +3 was considered as positive when there was a strong complete membrane staining in more than 10% of the tumour cells [16,17]. Staining for ER, PR, HER-2 and Ki-67 are shown in Figure 1. The tumours were classified into four biological subtypes: luminal A (ER + and/or PR+, HER2-), luminal B (ER + and/or PR+, HER2+), HER2-enriched (HER2+, ER-, PR-) and TN (negative for all three markers) [18].

### Ethical Approval

This research proposal was presented to the joint CUHAS-Bugando ethic and research committee where it received approval.

### Consent

This research is a retrospective study where histological slides, tissue blocks and patient’s files have been used to gather the information. The confidentiality of the patient was maintained at all time of the study. Only histology numbers and file numbers were used.

### Limitations

Being a retrospective study, patients were not followed up as they were referred to a cancer institute located far away from the study area for further treatment. Tissue blocks for immuno-stains were difficulty analyze due to poor storage, leading to evaluation of only 52 blocks from a total of 80 blocks selected. Statistical tests could not be validated due to limited number of cases.

## Results

The mean age at diagnosis of breast cancer was 49 years old. Four patients (7.7%) were below 30 years of age, whereas 10 (19.2%) were above 60 years. Fourteen patients (26.9%) were between 41 and 50 years of age, followed by 12 (23.1%) who were between 51 and 60 years and 25 (48.1%) above 50 years of age. Tumor size ranged from 2 cm to 12 cm in diameter with a mean tumor size of 5.9 cm, with half of the patients (50%) presenting a tumor size of more than 6 cm. Histological findings showed that the majority of the tumors were invasive ductal carcinoma 47 (90.4%), followed by invasive lobular carcinoma 3 (5.8%). One medullary carcinoma and one micropapillary carcinoma were diagnosed. The majority of the tumors 40 (76.9%) were of histological grade III. Thirty eight patients (73.1%) had lymph node metastasis and 36 (69.2%) were in clinical stage III, specifically IIIB. Only 3 patients (5.8%) were in clinical stage I. There was no patient with *in situ* carcinoma. There was a tendency for a low level of expression of the receptors, whereby ER positive tumors were 17 (32.7%), PR positive tumors were 22 (42.3%) and HER-2 positive tumors were 12 (23.1%). The proliferation index by KI-67 was high in 39 (75%) of the tumors. Based on the immuno-histochemical expression of the three markers, the proportion of breast cancer subtypes seen in this study were: Lumina A tumors 18 (34.6%), Lumina B tumors 9 (17.3%), HER-2 Enriched tumors 5 (9.6%) and Triple negative tumors 20 (38.4%). The majority of the patients were in a late clinical stage at the time of diagnosis and there was no difference between triple negative and non-triple negative patients. Triple negative tumors were seen more frequently in an age above 46 years with no significant difference compared to non-triple negative cancers. However, a small tumor size was more common in triple negative tumors compared to non-triple negative tumors with no significant difference and none of the TN tumors were in histology grade I. There was no difference in lymph node metastasis and proliferation index between triple negative and non-triple negative tumors (See Table 1).

**Table 1:** Clinico-pathological features of Triple Negative cancers and non-Triple negative cancers

		**TN cancers**	**Non TN cancers**	
	**N**	**n (%)**	**n (%)**	**p-value**
**Variable**				
**Age (years)**				
Less or equal to 46	24	8 (33.3)	16 (66.6)	
Above 46	28	12 (42.8)	16 (57.2)	0.339
**Tumour size**				
Below 6 cm	26	7 (26.9)	19 (73)	
Above 6 cm	26	13 (50)	13 (50)	0.077
**Histological grade**				
Grade I	3	0 (0)	3 (100)	
Grade II	9	3 (33.3)	6 (66.6)	
Grade III	40	17 (42.5)	23 (57.5)	0.325
**Lymph node metastasis**				
Yes	38	16 (42.1)	22 (57.9)	
No	14	4 (28.6)	10 (71.4)	0.288
**Proliferation index(Ki-67)**				
High	39	17 (43.6)	22 (56.4)	
Low	13	3 (23.1)	10 (76.9)	0.162
**Stage**				
Early stage	10	3 (30)	7 (70)	
Late stage	42	17 (40.5)	25 (59.5)	0.408

The majority of Lumina B tumors were large tumors of 6 cm and more, followed by triple negative tumors. Triple negative tumors showed a high proliferation compared to other subtypes with no significant difference. Lymph node involvement was more common in Triple Negative and HER-2 enriched tumors. Among patients below 50 years of age, 11 (61.1%) had lumina A subtype, followed by Lumina B 6 (66.6%) and triple negative was 9 (45%), (See table 2).

**Table 2:** Receptor status, subtypes and clinical pathological features

		**Cancer subtype**			
	**Lumina A**	**Lumina B**	**HER2 enriched**	**TN**	**p-value**
**Variable**	**n (%)**	**n (%)**	**n (%)**	**n (%)**	
Age below 50 years	11(61.1)	6(66.6)	1(20)	9(45)	0.282
Lymph node involvement	11 (61.1)	7(77.7)	4(80)	16(80)	0.568
Ki-67 high labelling	17(80.9)	4(44.4)	1(50)	17(85)	0.128
Tumour size above 6 cm	6(33.3)	7 (77.7)	5(100)	13(65)	0.009*

## Discussion

The demographic and clinical pathological features seen in this study were the same as described in two previous studies, whereas breast cancer was commonly seen in pre-menopausal women at an advanced stage with a high rate of lymph node metastasis and a large tumour size [19,20]. This study shows a tendency of a low expression of ER, PR and HER-2 in the selected patients; the same trend was observed in Dar-es-Salaam, although HER-2 was not tested [8], and in the neighbouring country of Kenya [9]. This shows therefore that only few patients in these settings would benefit from hormonal adjuvant therapy. A previous study, undertaken at the same health facility, showed that the modalities of treatment were mainly mastectomy with all the patients having been administered post-operative hormonal adjuvant therapy (tamoxifen). Adjuvant chemotherapy and radiotherapy was given in 44.8% and 11.7% of patients [20]. Since all the patients were given adjuvant hormonal therapy without testing the receptors, only 32% (ER+) benefited from this type of treatment. This shows that the majority of the patients were unnecessarily exposed to hormonal therapy which may be associated with side effects such as ischemic heart diseases, venous thrombosis, pulmonary embolism and endometrial cancer [21]. Studies have shown that there is no effect on breast cancer outcome on women with ER negative receptors when exposed to tamoxifen, whereas using of tamoxifen for more than 10 years in ER positive women with breast cancer leads to reduced recurrence and mortality [22].

In the current study, 23.1% of the patients were HER-2 positive, comprising the patients who fall under lumina B and HER-2 enriched tumours, so forming a subgroup of patients who can benefit from Trastuzumab. These findings did not differ from other studies which showed amplification of this oncogene to be in the range of 14-25% [4,6]. Patients with lumina B types can benefit from both hormonal therapy and trastuzumab, while patients with enriched HER-2 will benefit from trastuzumab only, as they are negative for hormone receptors. Lumina A tumors were found in 34.6% of the patients who could only benefit from hormonal therapy as they are negative for HER-2. Studies have shown that patients with positive ER had a much better response with trastuzumab than ER negative patients [23]; this includes patients with lumina B which comprised only 17.3% of our patients. Other studies demonstrated that the use of trastuzumab was associated with a longer time to occurrence of brain metastasis; even after brain metastasis, the use of this therapy showed a survival benefit [24].

Triple Negative breast cancers do not express any of the markers (ER, PR and HER-2). In the present study, 38.4% of the breast cancers fall into this category. These cancers were commonly seen in high grade tumours with a high rate of lymph node metastasis, large tumours size and tumours with necrosis. This type of breast cancers does not benefit from any of the neo-adjuvant targeted therapy. In general, TN tumours account for 10-17% of all breast cancers [5,13] and have been observed predominantly in patients of African ethnicity. This leads to the hypothesis that breast cancer is more aggressive in those patients. In studies undertaken in Western countries, breast cancer has proven to be more aggressive in Native Africans patients [15,25]. TN tumours tend to occur at a relatively young age with a more aggressive behaviour in African patients compared to Caucasians, presenting as large tumours, with a high proliferation rate and a high mortality [26]. The current study shows the same trend, whereby Ki-67 (proliferation marker) labelling was high in TN tumours, and these tumours were large and showed a tendency for a high rate of lymph node metastasis. Studies in Kenya found a prevalence of TN to be 44% (27). The modalities for neo adjuvant systemic therapy for TN tumours are either chemotherapy or radiotherapy with a high risk of recurrence, a high rate of brain metastasis and an overall poor outcome [28].

Furthermore, TN tumours are said to harbour a subtype of breast cancer called Basal Like breast Cancer (BLC). In this study, the marker for BLC was not investigated. The current study is the first study in Tanzania which addresses the TN tumours. In East African countries, Uganda and Kenya, BLC was found to range between 28 and 36% [27]. TN and BLC again form a subtype of cancers which harbour the carriers for BRCA-1 mutation [29]. Therefore there is high probability of detecting a number of patients who are carriers for BRCA-1 mutation in this setting. This is an area which needs further investigation, as molecular features of breast cancer have been shown to differ in various geographical locations and could explain the variability in aggressiveness and the disease outcome.

## Conclusion

This study showed a tendency for a low level expression of hormonal receptors and significant proportion of Triple Negative breast cancers with a high proliferation rate. It is recommended therefore to introduce routine testing for these markers before initiating hormonal therapy. Moreover, with the availability of Trasuzumub in Tanzania, some of the patients would be able to benefit from this targeted therapy.

## Abbreviations

BLC: Basal Like cancers; BMC: Bugando Medical Centre; DAB: Di-amino Benzidine; ER: Estrogen receptor; H&E: Hematoxylin and Eosin; HER-2: Family of epidermal growth factor receptor; IHC: Immunohistochemistry; PR: Progesterone receptor; TN: Triple Negative.

## Competing interests

The authors declare that they have no competing interests.

## Authors’ contributions

RP Involved in a study design, data collection, pathological review of H&E histological slides, analysis and preparation of the manuscript, MN involved in the preparation of the study, clinical data collection, manuscript preparation, CP involved in preparation of the study, clinical data collection, and preparation of the manuscript, JK Involved in preparation of the study, together with main author reviewed the histological slides and manuscript preparation, PS involved in reviewing the H& E histological slides and reading the immunohistochemical stains and FISH, BS performed all immunohistochemical stain and FISH, preparation of the manuscript. All the authors read and approved the final manuscript.
